# Effect of Preoperative Body Composition on Postoperative Anastomotic Leakage in Oncological Ivor Lewis Esophagectomy—A Retrospective Cohort Study

**DOI:** 10.3390/cancers16244217

**Published:** 2024-12-18

**Authors:** Jonas Herzberg, Tim Strate, Leon Passlack, Salman Yousuf Guraya, Human Honarpisheh

**Affiliations:** 1 Department of Surgery, Krankenhaus Reinbek St. Adolf-Stift, 21465 Reinbek, Germany; 2 Department of Internal Medicine, Krankenhaus Reinbek St. Adolf-Stift, 21465 Reinbek, Germany; 3 Clinical Sciences Department, College of Medicine, University of Sharjah, Sharjah P.O. Box 27272, United Arab Emirates

**Keywords:** Ivor Lewis esophagectomy, anastomotic leakage, esophageal cancer, sarcopenia, nutrition

## Abstract

Background: Surgery for esophageal cancer has an associated high rate of postoperative complications such as anastomotic leakage (AL) and fistulas. Pre-operative sarcopenia as a loss of skeletal muscle mass and function is identified as a potential prognostic factor in determining the outcomes of oncological surgical resections for esophageal cancers. In this study, we evaluated the impact of body composition on postoperative complications in esophageal cancer surgery. Methods: In this cohort study, we analyzed patients’ body composition at the level of the third lumbar vertebra on CT scans before Ivor Lewis resections for esophageal cancers between January 2015 and December 2022. Patients with a skeletal muscle index (SMI) ≤ 38.5 cm^2^/m^2^ in women and ≤52.4 cm^2^/m^2^ in men were classified as sarcopenic. Postoperative complications were categorized following the Dindo–Clavien classification and included AL, postoperative pneumonia, length of hospital stay, and failure-to-rescue which were compared between the sarcopenic and non-sarcopenic patients. Results: From a group of 111 patients with Ivor Lewis esophagectomy, 70 patients (63.1%) were classified as sarcopenic based on the SMI and the previously published gender-specific cut-off values. AL occurred at 12.6% (5.6% in adenocarcinoma). Within the whole cohort, patients with AL had a significantly low SMI of 43.487 ± 8.088 vs. 48.668 ± 7.514; *p* = 0.012. Additionally, the SMI showed a negative correlation to the length of postoperative hospital stay (r = −0.204; *p* = 0.032; *N* = 111). The failure-to-rescue rate was higher in the group of sarcopenic patients (12.8% vs. 8%). Conclusions: Our data showed a correlation between SMI and AL. This effect could not be seen in gender-specific SMI. This study showed a lower failure-to-rescue rate in non-sarcopenic patients after Ivor Lewis esophagectomy. These findings underscore the crucial role of determining the preoperative nutritional and body composition status as measured by the preoperative CT scans.

## Simple Summary

Esophageal cancer surgery is associated with a high risk of postoperative complications such as anastomotic leakage (AL). Preoperative malnutrition is a potential risk factor but the best way to diagnose its status remains unclear. The use of preoperative CT scans to detect deficits in body composition such as a reduced skeletal muscle index (SMI) can help to identify high-risk patients for postoperative complications. This study aimed to analyze clinical data of 111 patients after esophagectomy by correlating their preoperative CT scans with postoperative outcomes. These data showed reduced SMI in patients with postoperative (AL) and a prolonged postoperative length of stay in hospital in this group.

## 1. Introduction

Despite the advent of surgical innovations, esophageal oncological surgery is still associated with high mortality, and surgical resection remains to be the core element in the treatment of esophageal cancer [1]. The Ivor Lewis esophagectomy followed by a gastric tube reconstruction is a standard surgical technique for tumors in the distal esophagus and in the gastroesophageal junction. Even with new improvements in technique, instruments, and perioperative care [2], anastomotic leakage (AL) remains a significant complication after esophageal resection with a reported incidence of up to 30% [3,4]. A postoperative AL increases the risk of postoperative mortality with associated poor surgical outcomes [5,6].

Till now, the pathogenesis of AL remains unclear, whereas some risk factors like surgical technique and surgical experience have been detected [7]. Apart from these external factors, individual risk factors like diabetes or hypoproteinemia have been reported [8,9]. This underlines the importance of the preoperative metabolic status and the potential imbalance between energy stores and requirements [10,11,12]. The impact of the nutritional status has recently been underlined by Boccardi et al. [13]. The best way to evaluate the preoperative nutritional status is still under discussion and ranges from questionnaires, preoperative body mass index (BMI) to serum protein levels [14].

In this context, the terminology of sarcopenia becomes more important. This is originally a geriatric syndrome defined by muscle loss, reduced muscle strength, and dysfunction that also occurs in younger patients and is influenced by malnutrition and cancer [15]. Therefore, these conditions essentially lead to poor physical performance with high morbidity and mortality [16]. In several cancer types, a correlation between sarcopenia and increased postoperative morbidity has been shown already [15].

The loss of muscle mass and function in sarcopenia is commonly diagnosed using bio-impendence measurement and clinical tests such as hand-grip tests. Apart from this, the measurement of body composition and especially the skeletal muscle mass using CT scan was mentioned as a new technique in the current European Consensus Definition on Sarcopenia in 2019 [16]. The skeletal muscle index (SMI) as one of the body composition parameters can then be used to define a patient as sarcopenic as proposed by Prado et al. [17].

Till now, the clear correlation between preoperative body composition and postoperative major complications in other cancer types such as pancreatic adenocarcinoma [18] are not demonstrated in patients with esophageal cancer. Current data on esophageal cancer patients is inconsistent and clinical trials are essential to establish a correlation between body composition, calculated sarcopenia, and postoperative complications following oncological esophageal procedures.

On the one hand, in 2016, Grotenhuis et al. evaluated 120 esophageal cancer patients without any impact on postoperative short- or long-term outcome [19]. On the other hand, Fehrenbach et al. presented data from 85 locally advanced esophageal cancer patients showing a higher risk for postoperative pneumonia and a shorter long-term follow-up in sarcopenic patients [20]. Cossu et al. confirmed these findings and demonstrated a correlation between sarcopenia and postoperative AL and postoperative mortality in a multicenter study on esophageal cancer patients with an AL of 24.2% [3]. Apart from these parameters, the failure-to-rescue rate (FTR) can be considered an important overall outcome parameter in upper-GI cancer surgery as proposed by Busweiler et al. [21]. The impact of preoperative sarcopenia on the FTR is still unclear.

The aim of this study was to investigate the correlation between preoperative CT-based body composition and the occurrence of postoperative complications focusing on AL, postoperative pneumonia, and the FTR as core parameters in the postoperative short-term outcome.

## 2. Materials and Methods

All patients who underwent oncological surgical resections of the esophagus at Krankenhaus Reinbek St. Adolf-Stift, Germany between January 2015 and December 2022, were retrospectively evaluated. This study center is a certified center for esophageal cancer surgery. Patients with an Ivor Lewis resection, performed for malignant lesions of the esophagus or the esophagogastric junction were included in this analysis. Medical records of all eligible patients were reviewed and analyzed for the required data essential for the study. For all eligible patients, a routine preoperative work-up was performed, which included endoscopy, endosonography, and CT scan of the thorax and abdomen. After this work-up, all patients were reviewed in a multidisciplinary tumor board (MDT) meeting prior to surgery. A preoperative neoadjuvant treatment was considered in the MDT in accordance with the German national guidelines [22].

Medical records of the selected patients were screened for preoperative demographic and clinical parameters, surgical indications, history of smoking and tumor characteristics according to the pathological examination, which included tumor staging using the Union for the International Cancer Control (UICC) classification [23], multimodal treatment, BMI, and perioperative parameters. AL was defined as any radiological and/or endoscopically confirmed leakage of the anastomosis. The treatment of choice for AL was, depending on the size of the leakage, a re-operation, or the use of an endoscopic vacuum treatment. Postoperative pneumonia was defined as clinical presentations in combination with systemic inflammation marked by elevated leucocytes or fever postoperatively and a pathological radiological image such as infiltration in an X-ray or CT scan [24]. Complications were classified according to Dindo–Clavien’s classification [25] defining major postoperative complications as Grade >IIIa. As an additional outcome parameter, the FTR was evaluated [26,27]. This was calculated by dividing the number of deaths in patients with a postoperative complication by the number of patients with an adverse event.

All patients without malignant lesions or patients with segmental or transhiatal resection of the esophagus were excluded. Additionally, patients without preoperative CT scan or low-quality of preoperative CT scan were excluded.

### 2.1. Analysis of Sarcopenia

**Figure 1:**
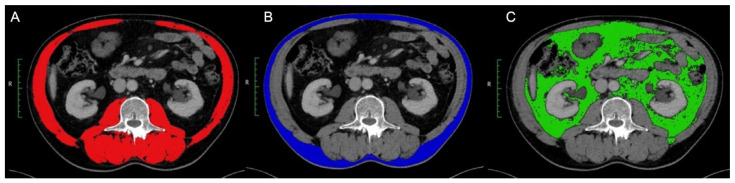
Detection of body composition within a CT scan at lumbar vertebra L3. (**A**) Total abdominal muscle area (TAMA) (red); (**B**) Subcutaneous fat tissue area (SFA) (blue). (**C**) Visceral fat tissue area (VFA) (green).

For the evaluation of body composition, preoperative CT scans performed within weeks before surgery were evaluated. In the case of neoadjuvant treatment, scans after the completion of the neoadjuvant therapy were used in accordance with the protocols proposed by by Mourtzakis et al. [28]. For each patient, a transverse slice plane was used in the portal–venous contrast phase at the level of the lumbar vertebral body 3. The Hounsfield units (HU) were used as markers for the tissues (Figure 1) [28].

The digital imaging and communications in Medicine (DICOM) dataset of the selected CT examination were transferred from the Picture Archiving and Communication System (PACS) to OsiriX (Pixmeo Sarl, Bernex, Switzerland) to analyze the CT. The region of interest (ROI) tool was used to semi-automatically determine the pixels with the defined HU. The measurement was checked and adjusted by a board-certified radiologist. Alongside the CT-based evaluation of the body constitution, the visceral fat tissue area (VFA) and subcutaneous fat tissue area (SFA) was also examined. The VFA was defined between −150 and −50 HU and the SFA between −190 and −30 HU as also reported by Cossu et al. [3]. The interval between −29 and +150 HU was used for the assessment of the total abdominal muscle area (TAMA).

The muscle area was adjusted to the body constitution by dividing it by the length squared calculating the SMI [cm^2^/m^2^]. A patient was classified as sarcopenic based on the definition presented by Prado et al., the SMI of <52.5 cm^2^/m^2^ in men and <38.5 cm^2^/m^2^ in women [17]. These cut-off values were also confirmed by Martin et al. [29] and used in several studies evaluating the body composition based on third lumbar vertebra CT scans [18,20]. If a BMI of >30 kg/m^2^ is also present, this is referred to as sarcopenic obesity [17]. The relationships between VFA, SFA, and TAMA (VFA/TAMA) as well as VFA and SFA were calculated and analyzed [20].

### 2.2. Surgical Technique

All patients in this study were treated by an abdominothoracic esophagectomy according to Ivor Lewis. As established in the center and in accordance with the MIRO trial, the resection was routinely performed at least as a hybrid approach, wherever possible [9,30]. This means a laparoscopic approach for gastric mobilization preserving the gastroepiploic vessels. After this minimal-invasive part, including a radical three-field lymphadenectomy, a thoracotomy, and an en-bloc transthoracic esophagectomy along with the mediastinal lymphatic node dissection was carried out. If possible, a complete minimally-invasive approach or a robotic approach was adopted. Within the study period, reconstruction was performed following two different techniques. The reconstruction was performed by a side-to-side anastomosis using linear stapling devices secured by additional sutures above the azygos vein. This surgical step was changed during the study period to an end-to-side stapled anastomosis with additional sutures due to a modification in the available stapling devices for applying circular stapling tools with three stapling lines.

During surgery, a nasogastric tube was introduced over the anastomosis and all patients were treated for at least 24 h in an intensive care unit depending on the clinical status. Oral intake began on the third postoperative day with clear fluids. 

All surgical procedures were performed by two experienced surgeons, who have completed their learning curve prior to our study.

### 2.3. Statistical Analysis

IBM SPSS Statistics Version 29 (IBM Co., Armonk, NY, USA) was used for statistical analysis. All linear variables were listed as means with standard deviations or as medians with minimums and maximums. Categorical variables were arranged as numbers with percentages. The chi-square test or Fisher’s exact test was used to determine relationships between categorical variables depending on group size. The Mann–Whitney-U-test was used to measure the probability of differences between two sets of data as a normal distribution could not be ensured in the study cohort. A *p*-value < 0.05 was considered statistically significant.

This study was conducted in accordance with the declaration of Helsinki after approval by the Ethics Committee of the Medical Association Schleswig-Holstein. This study was registered in the German Clinical Trial Registry (Study-ID: DRKS00029753).

## 3. Results

From January 2015 to December 2022, 118 patients were treated by Ivor Lewis resection due to esophageal malignancy at the study center. A total of 111 patients fulfilled the inclusion criteria, the majority of patients were men (98; 88.3%) with a median age of 65 years (35–88 years). In 89 patients (80.2%) an adenocarcinoma was diagnosed, whereas 20 (18.0%) patients had squamous cell cancer. Two patients (1.8%) showed other malignant lesions (Table 1). The CT scans were performed 2.85 ± 1.98 weeks prior to surgery.

In our study cohort, 70 patients were classified as sarcopenic based on their body composition and the defined threshold values (Table 1) [17]. The type of surgical reconstruction was equally distributed and showed no significant correlation to postoperative complications (*p* = 0.555).

No correlation was found between sarcopenia and postoperative complications (Table 2). In total, 12.6% patients developed AL with 12.9% in the sarcopenic and 12.2% in the non-sarcopenic group (*p* = 0.584). We found seven postoperative deaths in 64 patients with at least one postoperative complication, and there was an FTR of 10.9% in the overall cohort. This was mainly due to postoperative respiratory failure or AL. The FTR was 12.8% in the sarcopenic group, whereas this was only 8% in patients without preoperative sarcopenia, even so, this difference did not reach the level of statistical significance.

**Figure 2:**
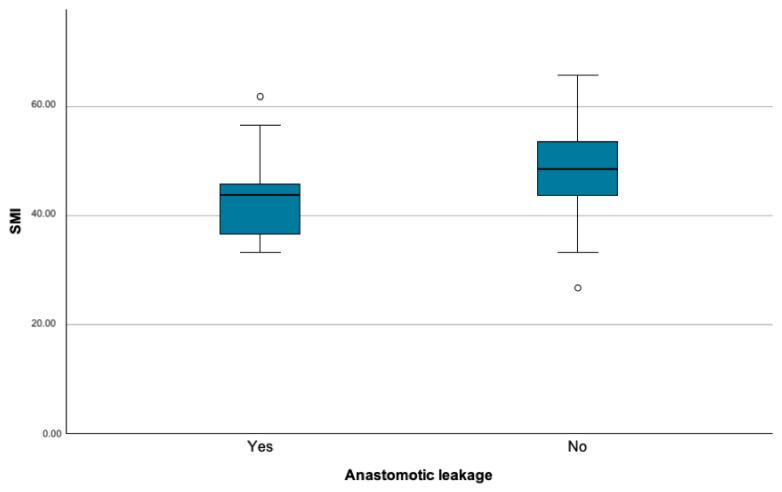
Skeletal muscle index (SMI) in patients with and without postoperative anastomotic leakage.

Comparing the raw body composition parameters, the SMI in the whole study cohort was significantly lower in the patients with an AL than in those without this complication (43.487 ± 8.088 vs. 48.668 ± 7.514; *p* = 0.012; Figure 2).

**Figure 3:**
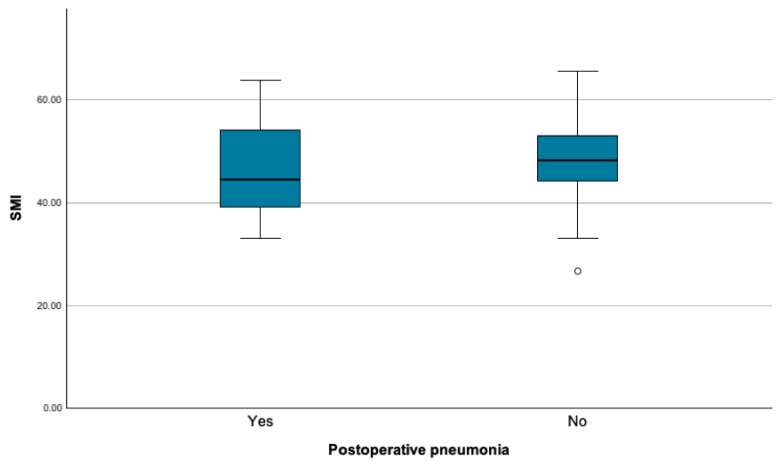
Skeletal muscle index (SMI) in patients with and without postoperative pneumonia.

This correlation could not be identified in patients with postoperative pneumonia (Figure 3) and after gender-specific differentiation (Table 3).

**Figure 4:**
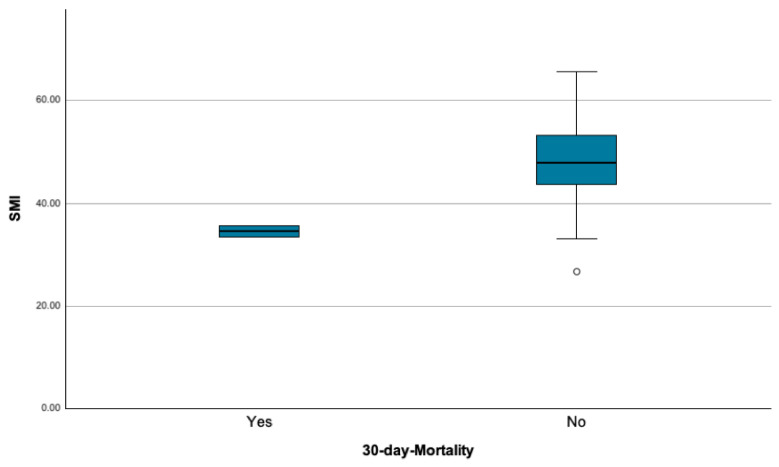
Skeletal muscle index (SMI) in patients with and without postoperative death within 30 days after surgery.

The SMI was significantly lower in the group of patients, who died within the first 30 days after surgery (34.583 ± 1.547 vs. 48.261 ± 7.595; *p* = 0.007; (Figure 4).

Additionally, the SMI showed a negative correlation with the length of stay (r = −0.204; *p* = 0.032; *N* = 111).

In addition, we evaluated the impact of the BMI on the outcome parameters mentioned. We could not identify any correlation for AL (no AL 26.91 kg/m^2^ vs. 25.02 kg/m^2^; *p* = 0.311), pneumonia (no pneumonia 26.98 vs. 25.95; *p* = 0.558) or postoperative 30-day mortality (no mortality *p* = 0.564

In patients with adenocarcinoma, postoperative AL was noted in 5.6% of all cases with no significant differences in BMI or SMI (Table 4). Combining squamous cell cancers and other malignant lesions, the AL rate is 40.9% higher showing a significantly lower SMI in the AL-group (41.830 ± 7.243 vs. 51.021 ± 3.769; *p* = 0.003).

## 4. Discussion

Our study evaluated the impact of preoperative body composition on postoperative complications after esophageal cancer surgery with an estimated AL rate of 12.6% following Ivor Lewis. Patients with AL had a significantly low SMI (43.487 ± 8.088 vs. 48.668 ± 7.514; *p* = 0.012). This was even more conspicuous in patients with non-adenocarcinoma (41.830 ± 7.243 vs. 51.021 ± 3.769; *p* = 0.003). This effect could not be found in gender-specific SMI evaluation. Sarcopenia, as defined by the well-established cut-offs used in this analysis, showed no influence on postoperative AL or pneumonia. Lastly, we found a higher FTR in the group of patients with sarcopenia (12.8% vs. 8%).

In our study, the rate of preoperative sarcopenia was found to be 63.1%, which is in line with the reported rates of 50% [31] to 85.6% [32]. These high rates of preoperative sarcopenia might be attributed to the tumor localization and associated manifestations of esophageal cancer such as dysphagia and malnutrition. In this context, preoperative nutritional status plays a major role in esophageal cancer surgery as highlighted in several studies [9,33]. Preoperative nutritional therapy carries a potential to reduce the risk of postoperative complications in esophageal cancer surgery [33,34]. Apart from biochemical parameters and BMI, body composition can be used as an additional parameter. This was evaluated in different studies for various oncological diseases including esophageal cancer [31,35,36]. The evaluation of body composition is routinely performed using dual-energy X-ray absorptiometry (DXA) measuring muscle mass, fat composition, and bone density [37]. Apart from that, CT or MRI are possible tools mainly used within trials showing a high correlation to measurements by DXA or body impedance measurement [38]. This is especially useful in case of routinely performed imaging such as in cancer patients [28,37]. Therefore, these imaging techniques for the routine evaluation of sarcopenia are newly recommended in the current European consensus definition of sarcopenia [16].

This approach is intensively studied using different techniques [16]. Several studies showed a positive correlation of the muscle mass at the third lumbar vertebra with the whole-body muscle mass [39,40,41]. Even so, this technique is associated with the limitation of measuring only the muscle mass and not the muscle strength. This technique has been evaluated in several studies to assess patients with sarcopenia [42,43]. After establishing this technique, cut-off values for sarcopenia based on calculated SMI are published [17]. Today, SMI cutoff values for men range from 52 to 55 cm^2^/m^2^ and for women from 39 to 41 cm^2^/m^2^ as analyzed in a published meta-analysis [40]. In this study, cut-off values of <52.5 cm^2^/m^2^ in men and <38.5 cm^2^/m^2^ were used to enhance comparability. These values were also used in recently published data from other centers [3,20].

The imperative role of body composition and sarcopenia in esophageal cancer surgery is still under discussion. Several studies evaluated the impact of preoperative sarcopenia on different postoperative parameters. Fehrenbach et al. reported a single-institution experience in locally advanced esophageal cancer [20]. In their study, the AL rate was 12.9% in adenocarcinoma patients, while our study had 5.6% AL in adenocarcinoma patients [20]. The authors of the study identified an elevated risk for major postoperative complications in patients with sarcopenia, whereas an association with AL could not be proven.

In their multi-center study, Cossu et al. showed an increased risk for 90-day mortality in patients with sarcopenia and documented a higher VFA/TAMA and VFA/SFA in patients with AL [3]. Paireder et al. reported a higher rate of conduit necrosis in their group of sarcopenic patients without influencing the overall AL rate [44]. The authors also reported a lower SMI in patients with squamous cell cancer of the esophagus without a correlation to the occurrence of postoperative AL [44].

In contrast to the studies showing an impact of preoperative body composition and sarcopenia on postoperative short- and long-term outcomes, some trials could not establish a scientific correlation. Grotenhuis et al. could not demonstrate any impact of sarcopenia measured by a preoperative CT scan on postoperative short- or long-term outcomes [19]. Grün et al. evaluated esophageal cancer patients before and after neoadjuvant treatment and the impact of SMI on postoperative short-term outcomes could not determine a significant correlation [45]. The authors attributed this finding to the intensive nutritional support at the study center but the overall rate of sarcopenia was 87.5% higher than reported in our cohort [45].

Our data showed a correlation between the overall SMI and the occurrence of postoperative AL, whereas no correlation could be established between body composition parameters and postoperative pneumonia, morbidity in general or mortality. Even then, the SMI was significantly lower in the AL group than in patients without AL; these values did not reach the cut-offs for gender-specific SMI or sarcopenia following the cut-off values proposed by Prado et al. in 2008 [17]. The importance of SMI as a core parameter for patients’ body composition was underlined by the use of this parameter in a prospective randomized trial evaluating the effect of a prehabilitation program during neoadjuvant treatment [46]. The importance of gender on patient’s body composition is well known [47,48], even so, current literature like the studies of Fehrenbach et al. or Cossu et al. do not report gender-specific body-composition parameters [3,20].

As sarcopenia not only refers to muscle quantity but also muscle quality, the evaluation of muscle strength as a quality parameter should be considered. Marano et al. evaluated the handgrip strength in a perioperative setting for abdominal surgery and showed that handgrip strength is inversely correlated with postoperative length of stay [49]. The muscle quality can also be defined using imaging tools such as CT scans measuring the muscle density [50]. This parameter should be measured using non-contrast-enhanced scans so evaluation of muscle density should be performed using native CT scans [51,52]. Singh et al. compared clinical tests for muscle strength with imaging techniques showing a high correlation between muscle strength and SMI [53].

Apart from all body composition parameters measured by CT or DXA, easily accessible parameters like BMI should be taken in mind in evaluating preoperative nutritional status. Our group has published data underlining the impact of preoperative hypoproteinemia in esophageal surgery [9]. As reported in the study in 2021, also in the present study no correlation between BMI and adverse postoperative events like AL could be found. The current literature shows different results. Zhu et al. describe a BMI > 24 kg/m^2^ as a risk factor for AL [54], whereas van Kooten et al. published a meta-analysis describing a BMI between 18.5 and 25 kg/m^2^ as a risk factor for postoperative mortality [55].

Apart from postoperative major complications such as AL and pneumonia, FTR is a powerful tool to measure the quality of care of patients postoperatively. The influence of sarcopenia on FTR has been evaluated in pancreatic cancer surgery and liver transplantation, which showed a higher FTR in sarcopenic patients [56,57]. In esophageal cancer surgery, little is known about the risk factors for FTR [58]. A large study by Abdelsattar et al., analyzing post-esophagectomy data from a large national American database, reported individual factors such as age or liver diseases and hospital volume [58]. At the hospital level, an FTR of 13.4% was reported in high-volume hospitals. A reported rate of 10.8% of FTR is below the commonly reported rate elsewhere [58]. At the same time, the effect of sarcopenia as an individual factor on FTR was not previously studied. In our study, comparing both groups, we found a lower FTR in patients without preoperative sarcopenia (8% vs. 12.8% in sarcopenic patients). Even though this difference did not reach the level of statistical significance, the trend is obvious. This is in line with the published data on the postoperative mortality of patients following oncological resections for esophageal cancers presented by Yunrong et al. in 2024, who reported a correlation between low preoperative psoas muscle index and a higher 30-day mortality [59]. The authors attribute this finding to a reduced tolerance to postoperative complications in patients with a lower muscle index [59].

## 5. Limitation

Although our study presents a relatively large number of patients, it is limited by its retrospective single-center cohort design evaluating a single time point. As sarcopenia not only refers to muscle mass but also to muscle strength, the additional evaluation of muscle function, for example, by measuring handgrip strength is needed even though this seems to have a high correlation with SMI [53]. Apart from known functional tests, this can also be carried out using CT scans measuring the density. Nevertheless, the body composition parameters are comparable to the current literature on this topic, a prospective evaluation of these patients including exercise and nutritional protocols, CT scans before and after neoadjuvant treatment and follow-up are essential to determine the effect of body composition in esophageal cancer treatment. This shortcoming might have partly influenced our study outcomes.

## 6. Conclusions

This study underpins the importance of preoperative body composition in Ivor Lewis esophagectomy for esophageal cancer. As the preoperative CT scan is commonly performed without additional patient burden or costs, it can provide valuable information to identify high-risk patients prior to surgery. The identification of preoperative sarcopenia would provide a pathway for rehabilitation programs in such patients. Further studies including functional tests such as handgrip strength are needed to evaluate the real impact of sarcopenia. Our study showed a correlation between overall SMI and AL. In addition, this study is the first evaluation of the effect of body composition on the failure-to-rescue rate in esophageal cancer surgery. The elevated failure-to-rescue rate in patients with preoperative low muscle mass highlights its significance during the perioperative period.

## Figures and Tables

**Table 1:** Demographic characteristics of patients with oncological Ivor Lewis esophagectomy in this study (*N* = 111).

Features	Total *N* = 111	Sarcopenia *N* = 70	No Sarcopenia *N* = 41	*p*-Value
Age, Median (min–max) [years]	65 (35–88)	67 (35–88)	63 (38–82)	0.068 ^a^
Sex, *N* (%)	0.150 ^c^			
Male	98 (88.3)	64 (91.4)	34 (82.9)	
Female	13 (11.7)	6 (8.6)	7 (17.1)	
ASA classification, *N* (%)	0.570 ^b^			
ASA 1	3 (2.7)	1 (1.4)	2 (4.9)	
ASA 2	54 (48.6)	33 (47.1)	21 (51.2)	
ASA 3	53 (47.7)	35 (50.0)	18 (43.9)	
ASA 4	1 (0.9)	1 (1.4)	0 (0.0)	
CCI ≥ 5	15 (13.5)	12 (17.1)	3 (7.3)	0.118 ^c^
BMI, M ± SD [kg/m^2^]	26.7 ± 5.1	25.6 ± 5.1	28.5 ± 4.5	<0.001 ^a^
Smoking, *N* (%)	34 (30.6)	22 (31.4)	12 (29.3)	0.493 ^c^
Pathology	0.247 ^c^			
Adenocarcinoma	89 (80.2)	58 (82.9)	31 (75.6)	
Other	22 (19.8)	12 (17.1)	10 (24.4)	
Preoperative treatment	0.462 ^c^			
FLOT	69 (62.2)	46 (65.7)	23 (56.1)	
CROSS	29 (26.1)	15 (21.4)	14 (34.1)	
None	11 (9.9)	8 (11.4)	3 (7.3)	
Other	2 (1.8)	1 (1.4)	1 (2.4)	
Anastomosis	0.555 ^c^			
End-to-side	57 (51.4)	34 (48.6)	23 (56.1)	
Side-to-side	54 (48.6)	36 (51.4)	18 (43.9)	
pTNM stage				
pT *	0.788 ^b^			
pT0	11 (9.9)	7 (10.0)	4 (9.8)	
pT1	17 (15.3)	10 (14.3)	7 (17.1)	
pT2	18 (16.2)	10 (14.3)	8 (19.5)	
pT3	57 (51.4)	37 (52.9)	20 (48.9)	
pT4	6 (5.4)	5 (7.1)	1 (2.4)	
pN *	0.417 ^b^			
pN0	55 (49.5)	31 (44.3)	24 (58.5)	
pN1	26 (23.4)	17 (24.3)	9 (22.0)	
pN2	20 (18.0)	15 (21.4)	5 (12.2)	
pN3	8 (7.2)	6 (8.6)	2 (4.9)	
R0 resection	106 (95.5)	67 (95.7)	39 (95.1)	0.538 ^c^
Number of resected lymph nodes	35.6 ± 13.3	35 ± 14.1	36.5 ± 11.9	0.614 ^a^
Length of surgery [min]	401.3 ± 77.7	405.3 ± 85.5	394.5 ± 62.7	0.564 ^a^
VFA (cm^2^)	191.9 ± 119.0	174.0 ± 115.2	222.4 ± 120.5	0.034 ^a^
SFA (cm^2^)	193.5 ± 110.1	177.5 ± 104.4	220.7 ± 115.5	0.037 ^a^
TAMA (cm^2^/m^2^)	152.8 ± 29.5	140.9 ± 22.0	173.0 ± 30.0	<0.001 ^a^
VFA/TAMA	1.2 ± 0.7	1.2 ± 0.8	1.3 ± 0.7	0.494 ^a^
VFA/SFA	1.1 ± 0.6	1.1 ± 0.7	1.1 ± 0.6	0.505 ^a^

M: mean, SD: standard deviation, ASA; American Society of Anesthesiologists., BMI; Body mass index. ^a^ Mann–Whitney-U-test. ^b^ Chi-Square-test. ^c^ Fischer’s exact test. * Including only 110 patients, as no TNM classification for melanoma is available. Values are reported as numbers (%) except where otherwise specified.

**Table 2:** Surgical outcomes within the first 30 days after surgery (*N* = 111). Patients classified sarcopenic, if SMI was <52.5 cm^2^/m^2^ in men and <38.5 cm^2^/m^2^ in women.

Features	Total *N* = 111	Sarcopenia *N* = 70 (64 Male; 6 Female)	No Sarcopenia *N* = 41 (34 Male; 7 Female)	*p*-Value
Postoperative complications according to DC	0.839 ^a^			
Grade 0	47 (42.3)	31 (44.3)	16 (39.0)	
Grade 1	0 (0.0)	0 (0.0)	0 (0.0)	
Grade 2	30 (27.0)	17 (24.3)	13 (31.7)	
Grade 3	21 (18.9)	13 (18.6)	8 (19.5)	
Grade 4	6 (5.4)	4 (5.7)	2 (4.9)	
Grade 5	7 (6.3)	5 (7.1)	2 (4.9)	
Major complication DC ≥ 3b	28 (25.2)	18 (25.7)	10 (24.4)	0.532 ^b^
30-day mortality	2 (1.8)	2 (2.9)	0 (0.0)	0.530 ^b^
Failure-to-rescue rate	10.9	12.8	8.0	0.695 ^b^
Anastomotic leakage	14 (12.6)	9 (12.9)	5 (12.2)	0.584 ^b^
Endoscopic vacuum treatment	13 (11.7)	8 (11.4)	5 (12.2)	0.565 ^b^
Pneumonia	34 (30.6)	21 (30.0)	13 (31.7)	0.507 ^b^
Length of stay (mean ± SD), days	20.6 ± 13.4	21.0 ± 13.5	19.9 ± 13.3	0.474 ^c^

DC: Dindo–Clavien classification. ^a^ Chi-Square test. ^b^ Fischer’s exact test. ^c^ Mann–Whitney-U-test. Values are reported as numbers (%) except where otherwise specified.

**Table 3:** Gender-specific CT-specific body composition for main postoperative complications after esophagectomy (*N* = 111).

Postoperative Complication						
	Male			Female		
Pneumonia	Yes (*N* = 31)	No (*N* = 67)	*p*-value	Yes (*N* = 3)	No (*N* = 10)	*p*-value
SMI	47.406 ± 8.368	49.966 ± 6.367	0.113	38.619 ± 4.473	39.647 ± 7.826	0.866
VFA (cm^2^)	206.096 ± 129.445	200.843 ± 115.175	0.870	112.930 ± 46.321	111.442 ± 94.544	0.866
SFA (cm^2^)	188.101 ± 113.591	191.358 ± 106.999	0.888	180.983 ± 87.683	227.951 ± 134.487	0.866
TAMA (cm^2^/m^2^)	151.725 ± 28.538	162.291 ± 22.308	0.051	101.437 ± 14.965	107.639 ± 20.541	0.612
VFA/TAMA	1.382 ± 0.934	1.223 ± 0.667	0.574	1.159 ± 0.620	1.022 ± 0.785	0.866
VFA/SFA	1.159 ± 0.761	1.133 ± 0.587	0.734	0.697 ± 0.270	0.470 ± 0.327	0.237
Anastomotic leakage	Yes (*N* = 9)	No (*N* = 89)	*p*-value	Yes (*N* = 5)	No (*N* = 8)	*p*-value
SMI	45.996 ± 8.448	49.476 ± 6.946	0.129	38.971 ± 5.496	39.684 ± 8.205	0.770
VFA (cm^2^)	234.494 ± 154.608	199.270 ± 115.670	0.442	132.256 ± 92.715	98.991 ± 81.902	0.558
SFA (cm^2^)	181.114 ± 119.156	191.260 ± 108.109	0.917	175.432 ± 73.180	243.163 ± 145.264	0.661
TAMA (cm^2^)	148.346 ± 28.814	160.021 ± 24.282	0.186	104.484 ± 17.455	107.285 ± 21.014	0.661
VFA/TAMA	1.579 ± 1.172	1.242 ± 0.708	0.428	1.229 ± 0.746	0.944 ± 0.745	0.661
VFA/SFA	1.291 ± 0.773	1.126 ± 0.632	0.526	0.749 ± 0.367	0.381 ± 0.197	0.143

SMI: skeletal muscle index. VFA: visceral fat tissue area. SFA: subcutaneous fat tissue area. TAMA: total abdominal muscle area.

**Table 4:** Subgroup analysis of anastomotic leakage among different histological subtypes (*N* = 111).

Anastomotic Leakage			
Adenocarcinoma (*N* = 89)	Yes (*N* = 5)	No (*N* = 84)	*p*-value
SMI	46.469 ± 9.507	48.304 ± 7.900	0.510
BMI	27.278 ± 2.382	26.890 ± 5.148	0.510
Other malignancies (*N* = 22)	*N* = 9	*N* = 13	*p*-value
SMI	41.830 ± 7.243	51.021 ± 3.769	**0.003**
BMI	23.759 ± 5.984	27.009 ± 4.285	0.096

SMI: skeletal muscle index. BMI: Body mass index. Bold values representing statistical significance regarding the Mann–Whitney-U-test.

## Data Availability

The data presented in this study are available on request from the corresponding author.
